# CovC-ReDRNet: A Deep Learning Model for COVID-19 Classification

**DOI:** 10.3390/make5030037

**Published:** 2023-06-27

**Authors:** Hanruo Zhu, Ziquan Zhu, Shuihua Wang, Yudong Zhang

**Affiliations:** 1 School of Computing and Mathematical Sciences, University of Leicester, Leicester LE1 7RH, UK; 2 School of Computer Science and Technology, Henan Polytechnic University, Jiaozuo 454000, China; 3 Department of Information Systems, Faculty of Computing and Information Technology, King Abdulaziz University, Jeddah 21589, Saudi Arabia

**Keywords:** randomized neural networks, deep random vector function linking, convolutional neural networks, image classification, COVID-19 infections, non-COVID-19 pneumonia patients

## Abstract

Since the COVID-19 pandemic outbreak, over 760 million confirmed cases and over 6.8 million deaths have been reported globally, according to the World Health Organization. While the SARS-CoV-2 virus carried by COVID-19 patients can be identified though the reverse transcription–polymerase chain reaction (RT-PCR) test with high accuracy, clinical misdiagnosis between COVID-19 and pneumonia patients remains a challenge. Therefore, we developed a novel CovC-ReDRNet model to distinguish COVID-19 patients from pneumonia patients as well as normal cases. ResNet-18 was introduced as the backbone model and tailored for the feature representation afterward. In our feature-based randomized neural network (RNN) framework, the feature representation automatically pairs with the deep random vector function link network (dRVFL) as the optimal classifier, producing a CovC-ReDRNet model for the classification task. Results based on five-fold cross-validation reveal that our method achieved 94.94%, 97.01%, 97.56%, 96.81%, and 95.84% MA sensitivity, MA specificity, MA accuracy, MA precision, and MA F1-score, respectively. Ablation studies evidence the superiority of ResNet-18 over different backbone networks, RNNs over traditional classifiers, and deep RNNs over shallow RNNs. Moreover, our proposed model achieved a better MA accuracy than the state-of-the-art (SOTA) methods, the highest score of which was 95.57%. To conclude, our CovC-ReDRNet model could be perceived as an advanced computer-aided diagnostic model with high speed and high accuracy for classifying and predicting COVID-19 diseases.

## 1 Introduction

### 1.1 COVID-19

On 30 January 2020, the World Health Organization (WHO) formally declared the outbreak of COVID-19 and upgraded the pandemic to a public health emergency of international concern (PHEIC). COVID-19, generally identified as coronavirus disease 2019, is a widespread contagious disease caused by severe acute respiratory syndrome coronavirus 2 (SARS-CoV-2). According to the epidemiological report from the WHO, over 760 million confirmed cases and over 6.8 million deaths have been reported globally since the beginning of the COVID-19 pandemic until 16 March 2023 [1].

The first people infected by the virus were reported in Wuhan City, Hubei Province, China, and it later spread rapidly across the world [2]. Several studies have confirmed that the COVID-19 virus is primarily transmitted via respiratory droplets and contact routes, resulting in direct human-to-human infection [3–5]. Virus transmission happens when people come into close contact (within 1 m) with a confirmed infected person who has respiratory symptoms such as coughing or sneezing, and their exposed mucosae and conjunctiva organ could become a potential receiver of the virus [6]. Common symptoms of COVID-19 include coughing, fever, loss of smell (anosmia), and taste (ageusia). Moreover, long-term consequences occur in post-COVID-19, such as weakness, general malaise, fatigue, cognitive impairment, etc. [7,8].

Diagnosing and detecting coronaviruses significantly contributes to outbreak control and further measures such as isolation and medical treatment. Currently, the mainstream virus detection technology is the reverse transcript–polymerase chain reaction (RT-PCR) test [9–11]. According to research from *The Lancet Infectious Diseases,* when pooled simultaneously, nasal and throat reach a high positive predictive value with an accuracy of 97% [12]. Another comparable detection approach is medical imaging, with different imaging modalities, like computed tomography (CT) and X-ray, being considered the most commonly used technologies [13–15]. Although medical imaging has been proven to have limited specificity in identifying COVID-19 (due to overlapping features in chest CT images such as those characterizing adenoviruses, influenza, H1N1, SARS, and MERS) [16], imaging requires more commonly available medical equipment and provides higher sensitivity than the RT-PCR test [17,18]. In addition, medical imaging can be used to confirm diagnostic results when positive-negative RT-PCR test results occur [19–21]. Evidently, medical images based on CT and X-ray scans remain highly valuable for COVID-19 disease diagnosis.

### 1.2 Pneumonia

Pneumonia is an infection that inflames or swells the tissue to create something akin to air sacs (also known as alveoli) in the human respiratory organs, specifically the lungs [22–24]. An annually reported 450 million people are infected with pneumonia worldwide, with over 4 million confirmed deaths [25,26]. Hence, it is vital to identify pneumonia at an early stage and further defeat it with prompt medical treatment.

Identifying the responsible pathogen is a crucial part of diagnosing pneumonia, but this is time-consuming and necessitates medical knowledge. Thanks to the rapid development of medical imaging technology, chest CT and X-ray have been proven to be reliable diagnosis approaches since lesions can be directly observed in images. Comparing common pneumonia patients with COVID-19 patients, different features can be captured in medical images. According to Zhao, et al. [27], COVID-19 infections (89.47%) were most commonly distinguished from common pneumonia (6.67%) in patients with ground-glass opacity and multiple mottling in their lung scans. Interestingly, the applicability of AI technology to the task of classifying COVID-19 patients and non-COVID-19 pneumonia patients is theoretical and evidence-based.

Furthermore, the multi-classification task could be more practical compared with binary classification. The reason for this could be that the RT-PCR test has already shown great capability to identify the SARS-CoV-2 virus carried by COVID-19 patients with high accuracy, but distinguishing COVID-19 from other lung diseases still mainly depends on the patient’s medical images. On the other hand, common symptoms between COVID-19 patients and pneumonia patients, such as productive or dry cough, chest pain, fever, and difficulty breathing, confuse the clinic diagnosis. An auto-detection AI system based on chest scans could provide computer-aided detection (CAD) algorithms even if patients have similar clinical symptoms. Hence, this research aims to develop a deep learning (DL) approach to classify COVID-19 patients, non-COVID-19 pneumonia patients, and normal cases that could be applied in practice.

The large volume of research on computer-assisted technology significantly contributes to diagnosing and detecting coronaviruses in clinical applications. Common challenges could be described as follows: (a) information loss occurs when deepening the neural network; (b) complex architecture leads to resource waste and time-assuming problems; (c) the network is limited to generalizing different tasks; (d) prediction accuracy remains to be improved. In this paper, we used a novel approach:

- ResNet-18 was selected as the backbone model, due to its superiority over the other six classic CNNs in the ablation experiment: AlexNet, VGG, ResNet-50, GoogleNet, DenseNet, and MobileNet.
- Compared with the traditional pre-trained CNN, randomized neural networks (RNNs) improve the model performance by replacing the last five layers of the tailored CNN as well as addressing the problem of computing resource waste thanks to their lightweight architecture.
- Our feature-based RNN framework, designed with an auto-selection algorithm, allows the most adaptive model obtained on various domains, which indicates the improvement in the generalizability of the model.
- Our novel CovC-ReDRNet obtains the feature representation from the tailored CNN as well as auto-selects the deep random vector function link network (dRVFL) as the optimal classifier according to our feature-based RNN framework; further, it feeds the feature representation directly to the dRVFL to construct the entire network. A good model performance based on five-fold cross-validation was achieved, with an MA sensitivity, MA specificity, MA accuracy, MA precision, and MA F1-score of 94.94%, 97.01%, 97.56%, 96.81%, and 95.84%, respectively.
- Compared with the other seven state-of-the-art (SOTA) methods (95.57%), our proposed model achieved the highest MA accuracy (97.56%).

This paper is structured as follows. Section 2 summarizes related work on classification tasks in the context of COVID-19. In Section 3, the material used for our research is introduced. In Section 4, the methodology of our proposed model is explained. In Section 5, the experiment results are compared and discussed. Finally, the conclusion is provided in Section 6.

## 2 Related Work

Classification tasks in the context of COVID-19 have become increasingly important as the pandemic continues to spread globally. Deep learning models have been applied to various classification problems related to COVID-19, including but not limited to diagnosis, severity assessment, and prognosis prediction. In this section, we highlight some of the recent developments in this field, discuss the challenges and limitations of the existing models, and further provide the motivation for our present research.

One of the earliest and most widely studied classification tasks in COVID-19 is the diagnosis of the disease. A number of studies have proposed deep learning models that can diagnose COVID-19 based on chest X-ray images and CT scans. In 2020, COVID-Net [28] boomed the application of deep learning for detecting COVID-19 cases from chest X-ray images. Additionally, the largest open access benchmark dataset of COVID-19-positive cases was generated, namely COVIDx, which comprises 13,975 chest X-ray images across 13,870 patient cases and is constantly expanding.

Subsequently, COVIDX-Net [29] was proposed to assist radiotherapists in automatically diagnosing COVID-19 based on chest X-ray images. The proposed framework included seven different architectures of deep convolutional neural networks (CNNs). Experimentally, a good performance was achieved by VGG-19 and DenseNet with F1-scores of 89% and 91% for normal and COVID-19 classes, respectively. More recent studies [30–34] supported deep learning approaches to learn discriminative patterns from chest X-ray images and CT scans as well as achieved high accuracy in COVID-19 detection tasks. The contributions and limitations of SOTA methods in the COVID-19 diagnosis task are analyzed in Table 1.

Another important branch in the COVID-19 classification task is the assessment of disease severity. The severity of COVID-19 can vary greatly from patient to patient, which indicates the importance of identifying patients who are at high risk of developing severe complications. For example, a multi-task vision transformer (ViT) that leverages a low-level chest X-ray feature corpus obtained from a backbone network to diagnose and quantify the severity of COVID-19 was proposed by Park, et al. [34]. The severity quantification performance of the proposed model was evaluated in terms of mean squared error (MSE) with a 95% confidence interval (CI) of 1.441 (0.760–2.122), 1.435 (1.195–1.676), and 1.458 (1.147–1.768) in three external datasets, respectively. Additionally, Goncharov, et al. [35] proposed a CNN-based network that leverages all available labels within a single model, which outperformed existing approaches and achieved a 97% Spearman correlation in severity quantification.

More advanced deep neural networks have been proposed based on various clinical and demographic factors for severity assessment [36–39]; CNNs and recurrent neural networks in particular have been applied to this task with promising results. The contributions of SOTA methods to the COVID-19 severity assessment task are highlighted in Table 2. Therefore, deep learning methods could be used to determine the prognosis of patients with COVID-19 and further guide clinical decision making.

A further remarkable application is the prognosis prediction of COVID-19, which refers to the prediction of the outcome of the disease, such as recovery or death. Prognosis prediction is imperative for clinical decision making and resource allocation, as well as for the development of effective treatments. A deep-learning-based study [40] demonstrated its potential to forecast the number of upcoming COVID-19 infections, and could thus significantly contribute to epidemic control. Four standard forecasting models were tested for predicting newly infected cases, deaths, and recoveries in the ten following days. Another study [41] pointed out the importance of prognosis prediction with the aim of triaging patients effectively; thus, mortality of COVID-19 patients was forecasted for one aspect of prognosis. Better performances were obtained using LASSO and linear SVM, with sensitivities of 90.7% and 92.0%, specificities of 91.4% and 91.8%, and area under the receiver operating characteristics curves (AUCs) of 96.3% and 96.2%, respectively.

More recently, several studies proposed various deep learning architectures for prognosis prediction [42–45], such as feedforward neural networks (FFNNs) and gradient boosting machines (GBMs), which showed that deep learning models could provide reliable predictions of patient condition, and further provide a deep understating of virology as well as aid in disease control. The contributions of SOTA methods to the COVID-19 prognosis task are highlighted in Table 3.

As mentioned above, deep learning technologies are effective in solving various classification tasks related to COVID-19, including diagnosis, severity assessment, and prognosis prediction. However, there have been a limited number of multi-category classification tasks developed. A multi-category classification task based on deep learning algorithms could be used to accurately diagnose COVID-19 and distinguish it from other respiratory illnesses such as the flu, pneumonia, and other viral infections. It is of considerable importance that the symptoms of COVID-19 are similar to those of many other respiratory illnesses, and misdiagnosis can cause serious consequences for both the patient and public health.

Some three-category classification frameworks that distinguish COVID-19 patients from pneumonia patients and normal cases have been proposed in recent years. Hussain, et al. [46] proposed a CNN-based model dedicated to COVID-19 diagnosis and classification, named CoroDet. A novel database, the COVID-R dataset, was constructed by merging and revising eight COVID-19 open sources, containing 7390 pulmonary images from 2843 COVID-19 patients, 3108 normal cases, and 1439 pneumonia patients. In their three-category classification experiments, the presence of the pulmonary lesion feature of COVID-19 disease in X-ray images was used to differentiate COVID-19 infection from non-COVID-19 pneumonia. CoreDet measured through sensitivity, specificity, precision, recall, F1-score, and accuracy, achieving a good performance based on the average of five-fold cross-validation, that is, 92.76%, 94.56%, 94.04%, 92.50%, 91.32%, and 94.20%, respectively.

Xu, et al. [47] proposed a novel approach for COVID-19 screening, distinguishing COVID-19 from other types of viral pneumonia, especially influenza-A viral pneumonia (IAVP), based on pulmonary CT images. A total of 618 CT images were obtained from three top hospitals in China, including 219 COVID-19 cases, 224 IAVP cases, and 175 normal cases. An advanced model was developed based on the classic ResNet-18 with a location attention mechanism, achieving an overall accuracy of 86.7%. The three different measurements considered, recall, precision, and F1-score, were 86.7%, 81.3%, and 83.9% in the COVID-19 group; 83.3%, 86.2%, and 84.7% in the IAVP group; and 90.0%, 93.1%, and 91.5% in the normal group, respectively.

However, the performance of this model leaves much to be desired. On the other hand, a specific framework was designed for an inner target that only tests on a fixed dataset, which limits the model generalization for different tasks. Hence, research aiming to develop a generalized deep learning framework with high accuracy for the related domain is critical.

## 3 Materials

### 3.1 Data Modality

X-rays rely on invisible radiation of electromagnetic energy to create images of internal tissues, bones, and organs on film or digital media. When the body receives X-rays, different parts of the tissues allow different quantities of radiation to pass through. The body’s soft tissues (such as blood, skin, fat, and muscle) permit the majority of X-rays to pass through, which show up as dark gray on film or digital media. Bones or tumors are more densely packed than soft tissues and allow very little radiation to pass through, appearing as white on the X-ray [48–50].

Chest X-rays are used to assist in diagnosis as they can indicate if there is a lesion feature of COVID-19 disease present or if another pulmonary problem is occurring. COVID-19 commonly leads to air sacs in the lungs filling with fluid, further producing bilateral peripheral opacities (normally observed as ground-glass opacities (GGOs) with areas of consolidation, being nodular or mass-like), with a lower lung distribution on patients’ chest CT images being the typical appearance of COVID-19 pneumonia [51–53]. Nowadays, X-ray machines are widely available in hospitals, and typical features can be observed with chest scans, which indicates that chest X-ray images are a powerful strategy in the early diagnosis of COVID-19.

### 3.2 Dataset

**Figure 1:**
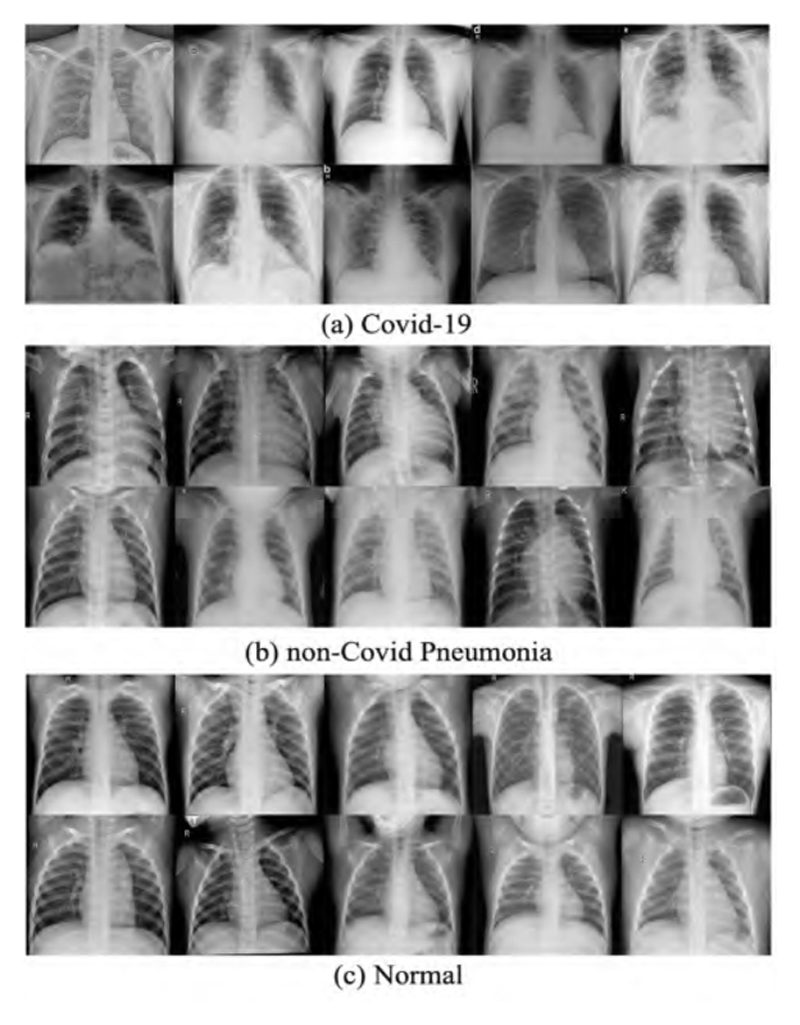
Templates for each category of the dataset.

Our proposed framework was implemented on the publicly available resource Kaggle, in the chest X-ray repository (COVID-19 & Pneumonia) [54]. The dataset is available at https://www.kaggle.com/datasets/prashant268/chest-xray-COVID19-pneumonia (accessed on 4 November 2022). The owner of the dataset, Prashant Patel, collected X-ray images from four different publicly available databases: a COVID-19 image data collection [55], pneumonia on chest X-rays [56], Figure 1 COVID-19 Chest X-Ray Dataset Initiative [57], and Actualmed COVID-19 Chest X-ray Dataset Initiative [58].

From the above open resources, 6432 chest X-ray images were collected. The training and test sets contained 5144 (80%) and 1288 (20%) images, respectively. The dataset is organized into three categories, namely COVID-19, non-COVID-19 pneumonia (including the viral and bacterial types of pneumonia), and normal (healthy cases). Table 4 summarizes the data distribution in different categories, and Figure 1 provides ten templates for each category of the dataset (the letter is determined by the X-ray machine but makes no relation to the classes).

## 4 Methodology

### 4.1 Selected Backbone Network

For our model design, a typical pre-trained CNN model was considered for the backbone of our proposed model, namely ResNet-18 [59]. ResNet variants show its significant dominance in computer vision fields, particularly to achieve a deeper network without expanding computational complexity, which is attributed to its exclusive architecture.

Generally speaking, a notable way to improve the performance of a model is to increase the depth of the network [60–63]. The deep CNN integrates features at different levels with the model in a layer-by-layer forward inference, which results in a more hierarchical and robust image feature, and subsequently, a better model performance.

However, gradient disappearance or gradient explosion is very likely to occur when deepening the neural network [64–67]. ResNet has an advanced framework designed to address this problem that adopts batch normalization (BN) right after each convolution and before activation. The underlying cause of gradient disappearance or gradient explosion is the erratic updating of network weights, essentially due to the multiplicative effect in gradient back-propagation. The normalization in ResNet can be divided into normalization from the beginning and internal normalization, which optimizes the stochastic gradient descent (SGD) used for back-propagation. The BN strategies unify the measure of captured features, making photographic features easier to expose and conduct; in other words, the addition of BN layers stabilizes the iteration of the network weights, hence allowing the deeper network to converge. For this reason, an approach that introduces ResNet with a BN layer to minimize disappearing or exploding gradients in deep neural networks could be considered.

On the other hand, deep neural networks suffer from a ‘degeneracy dilemma’ [68–71]. The degradation dilemma can be interpreted as a situation wherein the network with optimal performance is located in the shallow network but is not found by the SDG, with the consequence being that the deeper network underperforms the shallower network. A plausible explanation could be that each input-to-output procedure is almost irreversible due to the presence of the non-linear activation function (normally referred to as the ReLU), which inevitably results in significant non-reversible information loss. The designers of ResNet have proposed a mechanism to reduce the degradation problem by using identity mapping. The mechanism is capable of deepening the network in such a way as to ensure that the performance of the deep network is at least equal to that of the shallow network. However, current neural networks are incredibly challenging to fit into a potentially constant mapping function. An alternative solution is to learn the difference between the input and output. If the difference converges to zero, constant mapping can be obtained, thereby indirectly achieving constant mapping by fitting the residuals.

In mathematical conception, the residual is defined as the difference between the predicted and observed values. One block of residuals is described as

(1) $$x_{s+1}=x_{s}+\text{ℱ}\left(x_{s},w_{s}\right),$$

***x***_***s***+1_ refers to the predicted value after single-block residual learning, which can be interpreted as the output of the layer ***s*** + **1**. ***x_s_*** refers to the observed value before residual learning, which can be interpreted as the initial input of the layer $s.\text{ℱ}\left(x_{s},w_{s}\right)$ corresponds to the residual part of the *s*-layer network, which could be attributed to name this block as residual learning. Notably, the residual component is upgraded in Equation (2), which calculates the sum of each residual block when the residual learning module contains multiple residual blocks.

(2) $$x_{d}=x_{s}+\sum_{i=1}^{n}\text{ℱ}\left(x_{s},w_{s_{i}}\right),$$

where ***d*** ≥ ***s*** and $s\in {\mathbb{N}}^{+}$, indicating that the pattern from a shallower layer defined as ***x_s_*** could be mapped directly to a deeper layer defined as ***x_d_***; meanwhile, $\text{ℱ}\left(x_{s},w_{s_{i}}\right)$ corresponds to the residual part of the weight layer ***i***.

**Figure 2:**
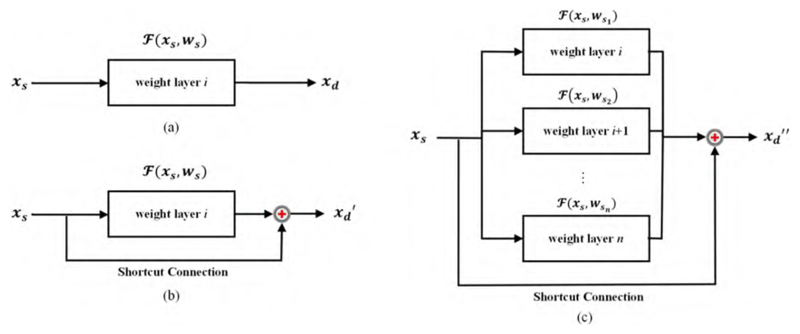
Comparison of residual learning with shortcut connections. Given the plain network (**a**), the single-block residual learning (**b**) is compared with the muti-block residual learning (**c**). In particular, ***x_s_*** refers to the patterns in shallow layers and ***x_d_*** refers to the deep layers. Notably, ***x_d_′*** remains in line with ***x***_***s***+1_ in Equation (1) and ***x_d_***″ with ***x_d_*** in Equation (2).

This paper chooses a ResNet with eighteen weight layers as the backbone of our model, referred to as ResNet-18. The structural diagram of a comparison of residual learning with shortcut connections is shown in Figure 2.

A residual-type connection is added to the original plain network, enabling the replication of features extracted at the shallow level to the additive deeper layer. It is a type of identification mapping that combines the features of the shallow network with the original plain stacking network output across one or more layers. The residual learning framework reduces the loss of information associated with deepening the network, thus achieving a better model performance.

Noteworthily, the deep system normally has a high training cost. If the system were to strengthen its training capability by simply stacking the plain layers, the number of parameters could exponentially explode. Understandably, training with tens of millions of parameters requires a GPU with a greater computational capacity, as well as a significant amount of training time. In addition, backtracking to the identity mapping algorithm, $x_{s+1}=x_{s}+\text{ℱ}\left(x_{s},w_{s}\right)$, it can be observed that the shortcut connection requires only one-step additive operations, whereas the parameters are trained by the plain network. Therefore, it is possible to improve the performance of the model with no additional parameters or costly computational complexity.

Furthermore, in our experimental section, ResNet-50 is used as the backbone for the control. On the other hand, another five CNN-based backbone networks are compared in our ablation experiments, namely AlexNet (2012) [72], VGG (2015) [73], GoogleNet (2015) [74], DenseNet (2017) [75], and MobileNet (2017) [76].

### 4.2 Tailored CNN

**Figure 3:**
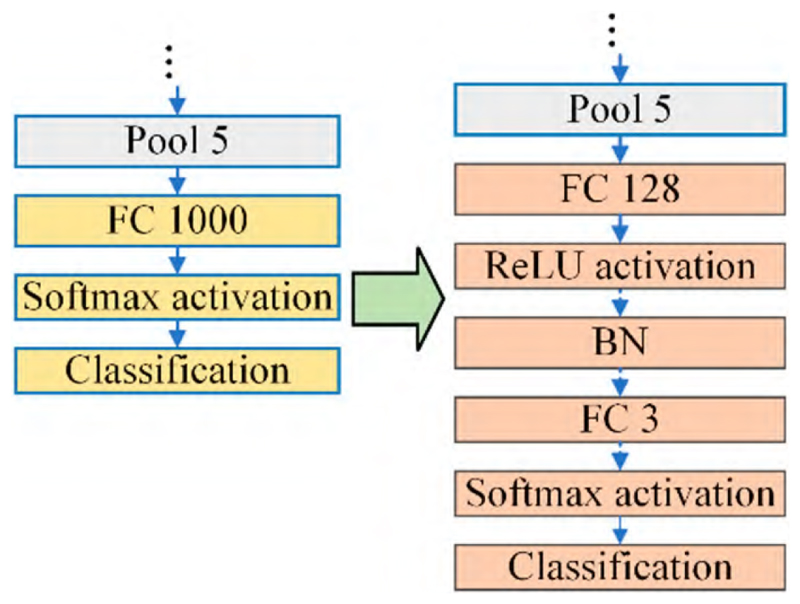
Tailoring the pre-trained ResNet-18.

The pre-trained ResNet-18 was selected as the backbone network of the proposed model. The CNN models that pre-trained on the ImageNet dataset acquired the ability to extract high-level image features. Therefore, the pre-trained CNN models could be extended to further image classification tasks according to the identified feature. However, some modifications should be made to the pre-trained ResNet-18 because of the difference between the ImageNet database and the public database used in this paper. The tailoring of the pre-trained ResNet-18 is presented in Figure 3.

Specifically, the ImageNet dataset has 1000 categories, but only 3 categories are referred to in this paper. Hence, ‘FC 1000’ is substituted with ‘FC 3’ because a three-class output is required for the group of COVID-19 patients, non-COVID-19 pneumonia patients, and normal cases. Further, the ‘FC 128’ layer is replaced with a ‘ReLU’ activation layer and a ‘BN’ layer is added for the purpose of mitigating the differences in dimensions between ‘Pool 5’ and ‘FC 3’. The resulting architecture of the tailored CNN is provided in Table 5.

### 4.3 Identified Feature Layer

**Figure 4:**
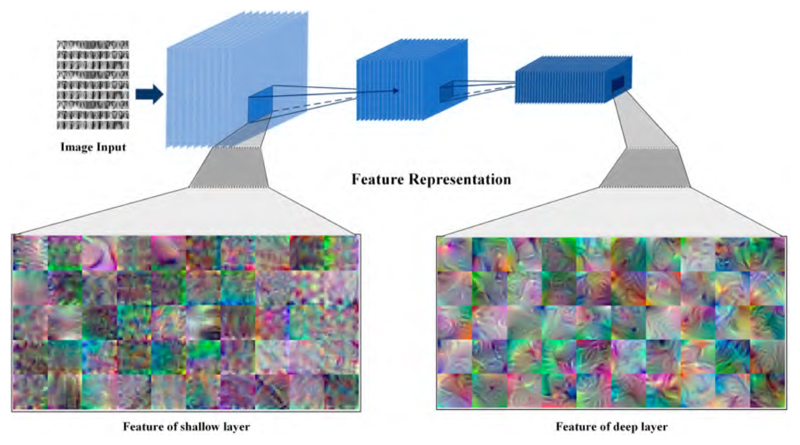
The procedure of the feature representation.

Feature extraction is a crucial step in deep learning, motivated by automatically learning informative and discriminative representations directly from the input data. In very recent years, pre-trained models derived from large-scale databases have been widely applied with great success to extract features for new tasks or new datasets. In particular, the feature representation procedure followed in our task is displayed in Figure 4.

Basically, the pre-trained models’ shallower layers typically learn low-level features, such as edges and corners, while higher layers learn more abstract and semantically meaningful features. According to conventional knowledge, the fully connected layer closest to the max pooling layer can capture the pattern of the picture to the greatest extent possible. Regarding the tailored CNN (Figure 3), the ‘FC 128’ layer following the last pooling layer was selected to be the feature layer for the following framework.

### 4.4 Feature-Based RNN Framework

Deep CNN models have achieved success in many areas. However, training deep CNN models is time-consuming because of the massive number of layers and parameters. In this paper, RNNs were selected to alleviate this problem because there are only single hidden layers with shallow architecture and non-single hidden layers with deep architecture. Moreover, the training of an RNN is often based on pseudo-inverse, which could contribute to fast convergence. Table 6 provides the mathematical symbol definitions.

#### 4.4.1 Shallow RNNs

**Figure 5:**
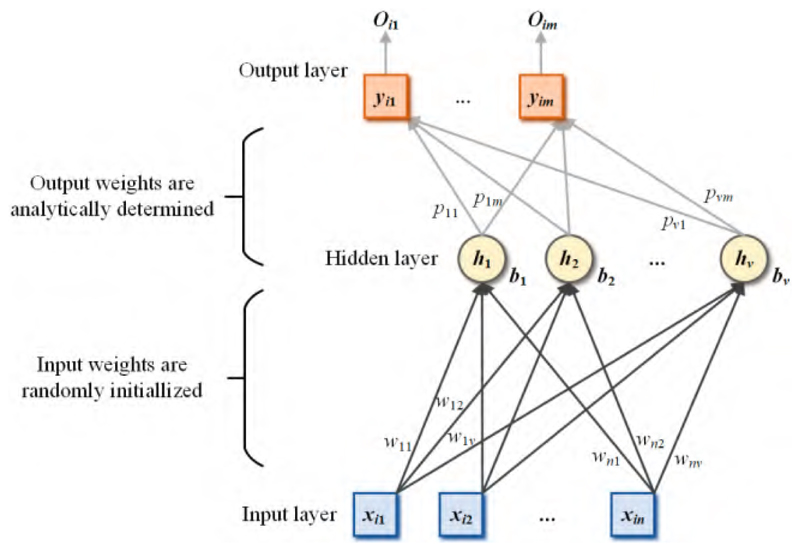
The structure of ELM.

Three leading RNNs, known as the extreme learning machine (ELM) [77], Schmidt neural network (SNN) [78], and random vector functional-link (RVFL) [79], were implemented initially within the model space of our proposed framework. In particular, the structure of the ELM is given in Figure 5.

For *N* arbitrary distinct samples, there is a training dataset, with its ***i***-th sample being

(3) $${\text{x}}_{\text{i}}={\left(x_{i1},\cdots ,x_{in}\right)}^{\text{T}}\in {\text{R}}^{n},i=1,\cdots ,N,$$

(4) $${\text{y}}_{\text{i}}={\left(y_{i1},\cdots ,y_{im}\right)}^{\text{T}}\in {\text{R}}^{m},i=1,\cdots ,N,$$

where *n* and *m* represent the input dimension and the output dimension, respectively. The original input matrix and the ground-truth label are presented as

(5) $$\text{X}={\left({\text{x}}_{1},\cdots ,{\text{x}}_{\text{N}}\right)}^{\text{T}},$$

(6) $$\text{Y}={\left({\text{y}}_{1},\cdots ,{\text{y}}_{\text{N}}\right)}^{\text{T}}.$$

The first calculation step of the ELM is given in

(7) $$M_{\text{ELM}\left(\text{i}\right)}=\sum_{j=1}^{\text{v}}\text{g}\left({\text{w}}_{j}{\text{x}}_{\text{i}}+{\text{b}}_{j}\right),\text{i}=1,\cdots ,N,$$

where *g*(·) is the activation function, ***w_j_*** is the weight which connects the input data with the ***j***-th hidden node, ***b**_j_* is the bias of the *j*-th hidden node, and *v* is the number of hidden nodes. The second calculation step is to calculate the output weight:

(8) $$\text{p}=M_{ELM}^{+}\text{Y},$$

where $M_{ELM}^{+}$ denotes the pseudo-inverse matrix of **M_ELM_**. Finally, the final output obtained as

(9) $${\text{O}}_{\text{i}}={\left({\text{O}}_{\text{i}1},\cdots ,{\text{O}}_{\text{im}}\right)}^{\text{T}}\in {\text{R}}^{m},i=1,\cdots ,N,$$

**Figure 6:**
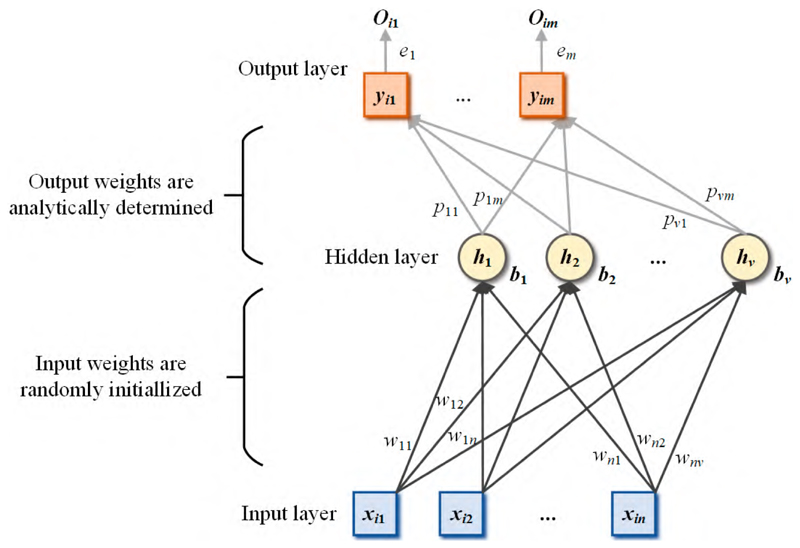
The etruraure of SNN.

The structure of the SNN is similar to that of the ELM, and the only difference is that there is a bias to the output layer in the SNN. The structure of the SNN is given in Figure 6. The output of the hidden layer is calculated as

(10) $$M_{SNN\left(i\right)}=\sum_{j=1}^{v}\text{g}\left({\text{w}}_{\text{j}}{\text{x}}_{\text{i}}+{\text{b}}_{\text{j}}\right),\text{i}=1,\cdots ,N.$$

The output weight is defined as

(11) $$\left(\text{p},e\right)=M_{SNN}^{+}Y,$$

where ***e*** refers to the output biases of the SNN.

**Figure 7:**
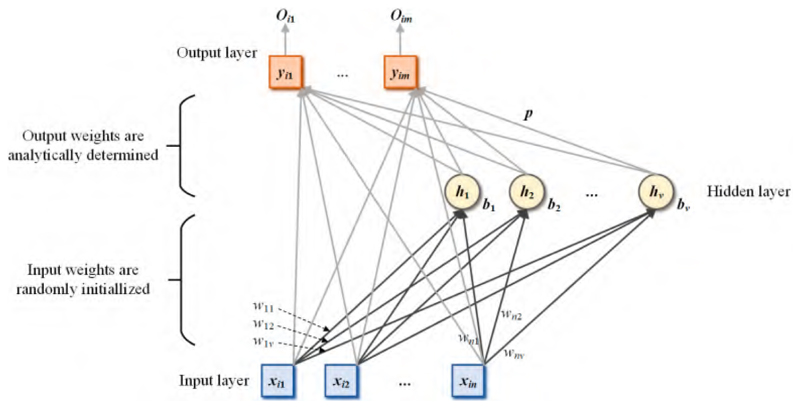
The framework of RVFL.

The structure of the RVFL is different from that of the ELM and SNN in that there are direct connections from the input layer to the output layer. The framework of the RVFL is presented in Figure 7. The calculation steps of RVFL are different and the output of the hidden layer is calculated as

(12) $$M_{\text{R}VFL\left(\text{i}\right)}=\sum_{j=1}^{v}g\left({\text{w}}_{\text{j}}{\text{x}}_{\text{i}}+{\text{b}}_{\text{j}}\right),i=1,\cdots ,N.$$

The input to the output layer is defined as

(13) $$D_{\text{R}VFL}=\text{concat}\left(\text{X},M\right),$$

where **X** represents the original input. The output weight is calculated as

(14) $$\text{p}=D_{\text{R}VFL}^{+}\text{Y}.$$

#### 4.4.2 Deep RNNs

**Figure 8:**
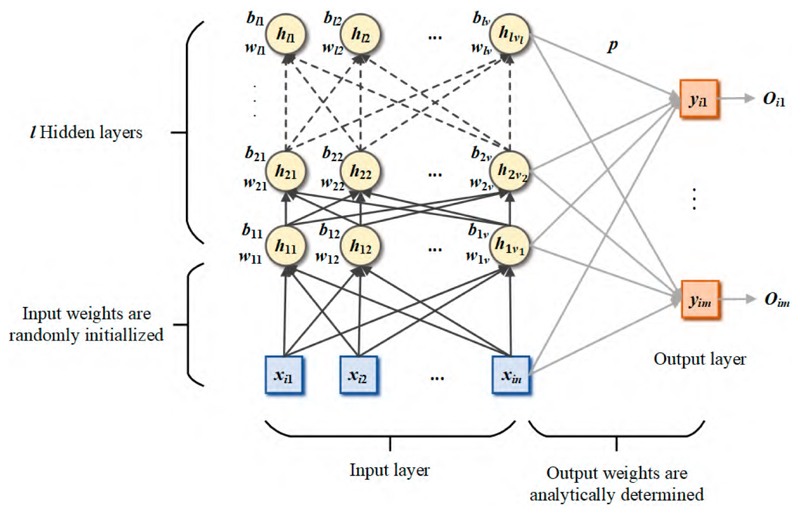
The framework of dRVFL.

The previous section detailed three shallow RNNs with a single hidden layer. However, it is not very stable because there are many randomization operations in the RNN. It is well known that deep architecture is more robust and accurate than a single neural network. Therefore, we increases the depth of the RNN based on the RVFL, namely the deep random vector function link network (dRVFL) [80]. The dRVFL functions as a high-speed automatic classifier attached to our feature extractor. It is characterized by stacked hidden layers, as shown in Figure 8.

In the dRVFL, the input of each hidden layer is the output of the previous layer. The input of the output layer is the ensemble of the output of each hidden layer and the original input. The calculation steps of the dRVFL can be defined as follows. The output of the first hidden layer is calculated as

(15) $$M_{dRVFL}{\;}_{i}^{1}=\sum_{j=1}^{v_{1}}g\left({\text{w}}_{1\text{j}}{\text{x}}_{\text{i}}+{\text{b}}_{1\text{j}}\right),i=1,\cdots ,N.$$

For *k* > 1, the calculation is defined as

(16) $$M_{dRVFL}{\;}_{\text{i}}^{\text{k}}=\sum_{j=1}^{{\text{v}}_{\text{k}}}g\left({\text{w}}_{\text{k}j}M_{dRVFL}{\;}_{i}^{k-1}+b_{kj}\right),i=1,\cdots ,N,k=2,\cdots ,l.$$

The calculation of the input of the output layer is performed as follows:

(17) $$D_{dRVFL}=\text{concat}\left(\text{X},M_{dRVFL}{\;}_{\text{i}}^{1},M_{dRVFL}{\;}_{\text{i}}^{2},\cdots M_{dRVFL}{\;}_{\text{i}}^{1}\right).$$

The output weight of dRVFL is given as

(18) $$\text{p}=D_{d\text{R}VFL}^{+}\text{Y}.$$

Notably, the deep architecture might not work for the SNN and ELM. A reasonable explanation for this could be that there is no interaction between the input layer and the output layer, whose instability could be augmented by more random weight in multiple hidden layers. Hence, only the dRVFL is added to the model space in our feature-based RNN framework.

#### 4.4.3 The Proposed Feature-Based RNN Framework

**Figure 9:**
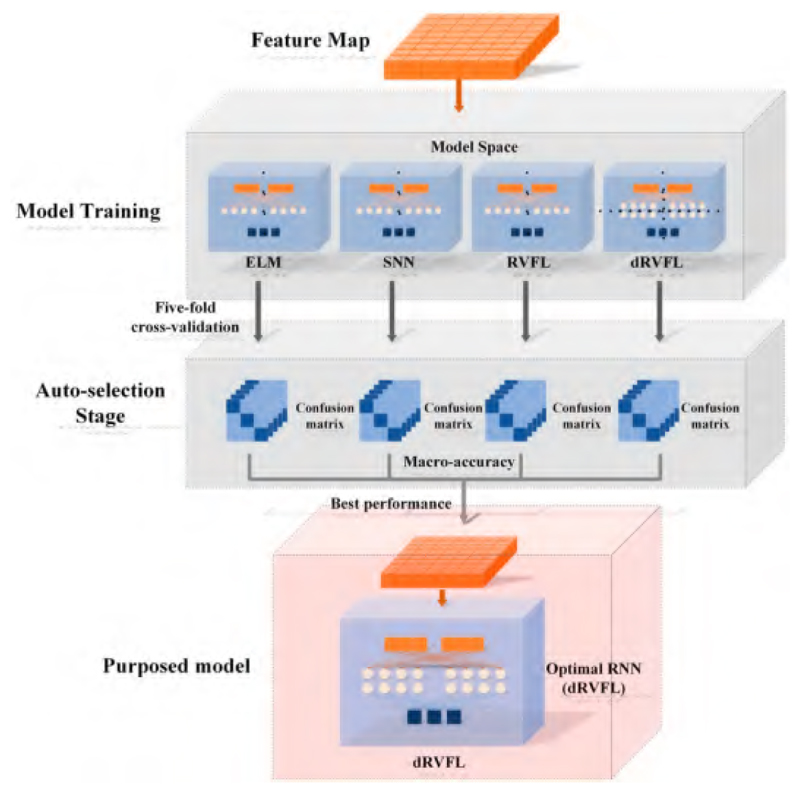
The structure of the feature-based RNN framework.

The fully connected layer ‘FC 128’ is identified as the feature map for the customized feature extractor. The selected feature is fed directly to our novel RNN framework for the specific classification procedure, namely, the feature-based RNN framework. Figure 9 shows the intuitionistic structure of our proposed RNN framework. The model space consists of three shallow RNNs and one deep RNN. Each RNN pairs with the selected feature map separately, for the purpose of exploring the prior classifier. The RNNs are evaluated mainly considering classification accuracy, with one optimal RNN as the output of the feature-based RNN framework. Further, the best classifier is the one that achieves the best feature extractor.

### 4.5 The Proposed Model

A novel model is proposed to distinguish COVID-19 patients from non-COVID-19 pneumonia patients as well as normal cases, abbreviated as the ResNet-18-based dRVFL network for COVID-19 classification (CovC-ReDRNet). A ResNet-18 pre-trained on the ImageNet dataset is introduced as the backbone model. Referring to the BN layers, ResNet-18 addresses the problem of gradient disappearance or gradient explosion. Moreover, the residual learning framework reduces the ‘degeneracy dilemma’ by using identity mapping, reducing the loss of information when increasing the depth of the network. The shortcut connection requires only one-step additive operations, indicating no additional parameters or costly computational complexity, despite the improvement in model performance.

The pre-trained ResNet-18 are necessary tailored according to the difference between the ImageNet database and the public database used in this paper. The layer ‘FC 1000’ is substituted with ‘FC 3’ because only three classes of output are involved rather than one thousand categories in our classification task, in particular, the COVID-19 patients, non-COVID-19 pneumonia patients, and the normal cases, respectively. Further, the ‘FC 128’ layer is replaced with a ‘ReLU’ activation layer, and a ‘BN’ layer is added with the aim of mitigating the differences in dimensions between ‘Pool 5’ and ‘FC 3’. The tailored pre-trained ResNet-18 is presented in Figure 3, and the entire architecture of the tailored CNN is provided in Table 5.

RNNs as classifiers stand out thanks to their ability to alleviate the time-consuming problem in traditional deep CNN models. This is mainly due to the streamlined architecture of RNNs, which only employ three shallow layers or five deep layers rather than massive layers and the parameters of CNNs. Additionally, special random nodes in hidden layers conduct the pseudo-inverse when training an RNN, which could contribute to the fast convergence. Three advanced shallow RNNs are implemented initially within the model space of our proposed framework, known as the ELM, SNN, and RVFL. Additionally, deep architecture, that is, the dRVFL, is involved in our framework to enhance robustness and stability compared to shallow RNNs. In the end, a feature-based RNN framework was used to evaluate the performance of the above classifier and eventually led to the development of the proposed model for the classification task.

**Figure 10:**
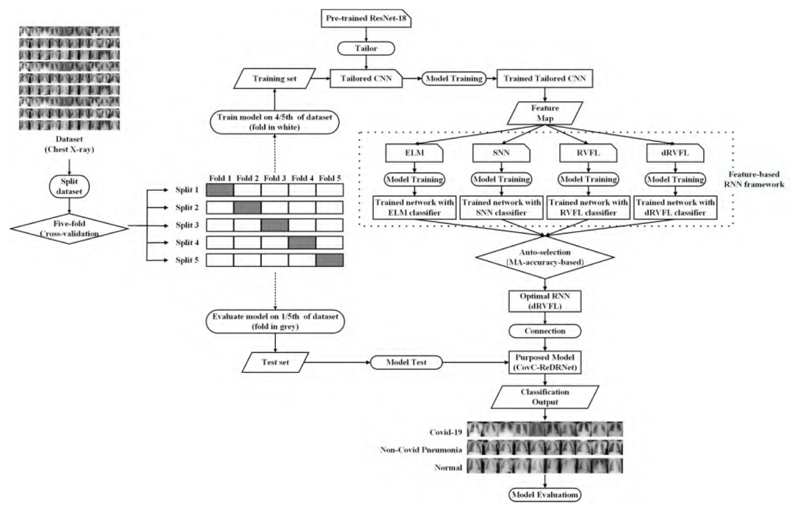
A diagram of the proposed CovC-ReDRNet model.

The proposed model was implemented with the output of the ‘FC 128’ layer as the optimal feature representation as well as the dRVFL as the optimal classifier. The classifier is selected with the auto-selection algorithm, which is advantageous in the identification of the best option while saving time and resources as well as ensuring consistent and fair decision making. A brief diagram is illustrated in Figure 10, and the pseudo-code is presented in Algorithm 1.

Furthermore, our novel auto-selection algorithm and feature-based RNN framework can be used to design the most adaptive model for a specific task. This demonstrates that our proposed framework maintains tremendous potential for improving the generalizability of a model, which could be adapted to more tasks in various domains.

### 4.6 Evaluation

Five-fold cross-validation is chosen to evaluate the performance of our proposed network. Five measurements are employed in this paper: accuracy, sensitivity, specificity, precision, and F1-score. These measurements for the classification of two categories are modified because there are three categories in this paper. When one category is defined as positive, the other two categories are set to negative. These measurements can be computed by

(19) $$\text{Accuracy}\left(c\right)=\frac{\text{TP}\left(c\right)+\text{TN}\left(c\right)}{\text{TP}\left(c\right)+\text{TN}\left(c\right)+\text{FP}\left(c\right)+\text{FN}\left(c\right)},$$

(20) $$\text{Sensitivity}\left(c\right)=\frac{\text{TP}}{\text{TP}\left(c\right)+\text{FN}\left(c\right)},$$

Algorithm 1 **The algorithm of the CovC-ReDRNet.**

**Algorithm** CovC-ReDRNet

**Input:** dataset *D*

**Output:** the classification performance *S* of the trained CovC-ReDRNet

1.      Import the original dataset *D*

2.      Implement five-fold cross-validation, split the dataset into five equally sized sets {*D*_1_, *D*_2_, *D*_3_, *D*_4_, *D*_5_}

3.      **For** each in {*D*_1_, *D*_2_, *D*_3_, *D*_4_, *D*_5_} **do**

4.          data.test = *D_i_*

5.          data. train = *D* – *D_i_*

6.          Load pre-trained ResNet-1 8 model *M*

7.          Remove the last three layers FC 1000, softmax, and classification layer from the *M*

8.          Replace with another six layers: FC128, ReLU, BN, FC 3, softmax, and classification layer

9.          Train the tailored CNN model *T* on data.train

10.        Set ‘FC 128’ following the last pooling layer as the feature layer

11.        Target the output of the feature layer as identified pattern ***i***

12.        Fed the ***i*** into our feature-based randomized neural network (RNN) framework *R*

13.        Compete the RNNs in {*R*_1_, *R*_2_, *R*_3_, *R*_4_} the ELM, SNN, RVFL, and dRVFL

14.        Select the optimal automatically according to the MA accuracy from the confusion matrix

15.        Connett the optimal classifier with the feature layer

16.        Construct the entire architecture of the proposed network, namely CovC-ReDRNet

17.        Test the troined CovC-RoDRNet on the data.test

18.      **End For**

19.      Report the classification performance of the trained CovC-ReDRNet

(21) $$\text{Specificity}\left(c\right)=\frac{\text{TN}\left(c\right)}{\text{TN}\left(c\right)+\text{FP}\left(c\right)},$$

(22) $$\text{Precision}\left(c\right)=\frac{\text{TP}\left(c\right)}{\text{TP}\left(c\right)+\text{FP}\left(c\right)},$$

(23) $$\text{F1}-\text{score}\left(c\right)=2\times \frac{\text{Sensitivity}\left(c\right)\times \text{Precision}\left(c\right)}{\text{Sensitivity}\left(c\right)+\text{Precision}\left(c\right)},$$

where *c* represents the category in this paper, and TN, TP, FP, and FN denote true negative, true positive, false positive, and false negative, respectively.

To ensure a comprehensive evaluation, macro-averaging is introduced to measure the overall system, namely MA accuracy, MA sensitivity, MA specificity, MA precision, and MA F1-score, respectively.

(24) $$\text{MA}\;\text{accuracy=}\frac{1}{n}\Sigma_{i=1}^{n}\;\text{Accuracy}\left(c_{i}\right),$$

(25) $$\text{MA}\;\text{sensitivity =}\frac{1}{n}\Sigma_{i=1}^{n}\;\text{Sensitivity}\left(c_{i}\right),$$

(26) $$\text{MA}\;\text{specificity}=\frac{1}{n}\Sigma_{i=1}^{n}\;\text{Specificity}\left(c_{i}\right),$$

(27) $$\text{MA}\;\text{precision =}\frac{1}{n}\Sigma_{i=1}^{n}\;\text{Precision}\left(c_{i}\right),$$

(28) $$\text{MA}\;\text{F1}\;–\;\text{score}\;\text{=}\;\frac{1}{n}\;\Sigma_{i=1}^{n}\;\text{F1–score}\left(c_{i}\right),$$

where *n* represents the number of categories in the experiment, and ***i*** = 1, 2, 3 stand for the categories of COVID-19, non-COVID-19 pneumonia, and normal cases, respectively.

## 5 Experiment Results and Discussions

### 5.1 Experimental Settings

The hyper-parameter setting is provided in Table 7. The maximum value of the epoch is set as 4 for the purpose of reducing the overfitting problem. The minimum value of the batch size is decreased to 10 due to the small size of the training set, in which only 5144 images are included. Based on convention, the learning rate is set to 10^−4^. In terms of RNNs, 400 is an appropriate value for the number of hidden nodes according to the input dimension; thus, the number of hidden layers in the dRVFL is set as 4.

### 5.2 The Performance of CovC-ReDRNet

Five-fold cross-validation was implemented to evaluate the proposed model. The MA accuracies of five-fold cross-validation are shown in Table 8. It was revealed that the MA accuracy of each fold was greater than 97% and the average achieved 97.56%, which is outstanding because accuracy is regarded as one of the most significant indicators for clinical diagnosis. We used five other indicators to comprehensively evaluate the proposed model in three categories: MA accuracy, MA sensitivity, MA specificity, MA precision, and MA F1-score.

The results of these indicators for the three classes are given in Table 9. Typically, the accuracy for the COVID-19 group reached 99.44%, which indicates that CovC-ReDRNet could be an extraordinary network for distinguishing COVID-19 patients from non-COVID-19 pneumonia patients or normal cases. Moreover, all results achieved by the proposed model were greater than 90%. It can be inferred that the proposed model could be a good choice for COVID-19 diagnosis.

In addition, a series of ablation experiments were designed for the purpose of comparing our proposed model with relative architectures. In the following ablation experiment, five-fold cross-validation was consistently applied, and the same measurements are used for comparison.

### 5.3 Ablation Study

#### 5.3.1 Superiority of ResNet-18 over Different Backbone Networks

This section contains two sections; for ablation experiment I, we compared ResNet-18 against six classic CNNs, AlexNet, VGG, GoogleNet, DenseNet, and MobileNet, respectively. For ablation experiment II, ResNet-18 competed with another ResNet variant, namely, ResNet-50.

The MA accuracies achieved in ablation experiment I are provided in Table 10. The average MA accuracies when using AlexNet, VGG, GoogleNet, DenseNet, MobileNet, and ResNet-18 (ours) backbones are shown in the last column, that is, 95.28%, 92.32%, 96.71%, 97.27%, 97.02%, and 97.56%, respectively. Our ResNet-18-based network, CovC-ReDRNet, achieved the highest MA accuracy among the six different backbones, which indicates that ResNet could provide a high-accuracy performance as our backbone model.

Additionally, according to Table 11, the performance is further discussed for COVID-19, non-COVID-19 pneumonia, and normal categories. In the COVID-19 group, the highest sensitivity and specificity were achieved by DenseNet-based and VGG-based networks, at 95.48% and 99.86%. Notedly, our model achieved the best performance in accuracy, precision, and F1-score at 99.44%, 98.40%, and 96.82%, respectively. Although the ResNet-18-based model did not have the best sensitivity and specificity, a slight difference of 0.17 and 0.01 percentage points could be observed. In both the non-COVID-19 pneumonia group and the normal group, CovC-ReDRNet defeated the other five backbone frameworks, achieving the most outstanding result in sensitivity, specificity, accuracy, precision, and F1-score.

For ablation experiment II, the MA accuracies and the measurements are elaborated in Tables 12 and 13, accompanied by the top score. It was found that our CovC-ReDRNet model achieved a better performance with all the indicators, which might be explained by the information wastage caused by the deep layers.

#### 5.3.2 Superiority of Deep RNNs over Traditional Classifiers

Compared with traditional transfer learning, our CovC-ReDRNet model outperformed the traditional classifier, the softmax modules typically used for ResNet-18, with a deep RNN classifier, and the dRVFL modules typically constructed in our novel model. In ablation experiment III, the different traditional classifiers were associated with their RNNs. In total, thirty architectures including our CovC-ReDRNet model were employed in this ablation experiment; that is, six pre-trained CNNs were modified with the transfer learning mechanism as well as with the RNN framework separately.

Along with the highest score, the MA accuracies based on five-fold cross-validation are presented in Table 14. It was found that the dRVFL technology was in the lead during the whole race. In particular, the dRVFLs stand out as basic classifiers, achieving 2.08, 3.22, 0.63, 0.15, 1.18, and 0.32 percentage points with the baseline of AlexNet, VGG, GoogleNet, DenseNet, MobileNet, and ResNet-18 (ours), respectively. According to the result, the dRVFL increases the MA accuracy significantly, which supports the superiority of RNNs over traditional classifiers. Moreover, our CovC-ReDRNet model had the top score among the thirty networks, which indicates the dRVFL framework demonstrates a greater capability of providing a more accurate algorithm for the COVID-19 classification task.

#### 5.3.3 Superiority of Deep RNN over Shallow RNNs

According to Section 4.4.1, the deep RNN had better stability and robustness compared with single-layer RNNs. Ablation experiment IV was designed for investigating the capability of the deep RNN over the single-hidden-layer architecture, in particular, the dRVFL architecture in our framework.

In Table 15, the dRVFL architecture evidences its superiority by exceeding all three single-hidden-layer networks, which supports that our RVFL deepening strategy significantly improves the MA accuracy. Additionally, the RVFL comes out on top in the competition of all shallow RNNs, obtaining an MA accuracy of 97.37% and defeating the ELM and SNN, which obtained accuracies of 96.52% and 96.60%, respectively.

Regrettably, both the deep extreme learning machine (dELM) and deep Schmidt neural network (dSNN) achieved a limited accuracy of 77.62%. The unsatisfactory model performance implies that the model fails to reflect proper disease classification, which could be explained by the network structure of the dELM and dSNN. There is no direct correlation between the ELM and SNN input and output layers; therefore, the random weights added to multiple hidden layers cause a significant loss of image pattern in classification. Accordingly, the experimental results for dELM and dSNN are worse than those for the ELM and SNN with a single hidden layer. Furthermore, the equivalent results signify that multiple hidden layers expand the impact of random weights on the classification performance much more than an output layer that is identical to the SNN. Therefore, using an RVFL combined with a deepened component, that is, dRVFL, could be a sensible approach.

The measurements for different classes in ablation experiment IV are described in Table 16, accompanied by the highest score. It can be observed that our CovC-ReDRNet model achieved the best performance with almost all indicators, but scored marginally lower than the RVFL on sensitivity in the non-COVID-19 pneumonia group by 0.12 percentage points as well as on specificity in the normal group by 0.06 percentage points.

### 5.4 Comparison Study

Comparison studies play a crucial role in research, helping to advance the research in a particular field by identifying the latest and best models. In this section, the cutting-edge deep learning research related to COVID-19 disease diagnosis and classification, especially that on the three-category classification task, is compared with our study. Table 17 shows the performance of models based on the level of their knowledge and provides a longitudinal digital comparison with our proposed model. It was found that our proposed model achieved the highest MA accuracy, achieving 97.56% accuracy, compared with the state-of-the-art (SOTA) methods with their score of 95.57%. Hence, this indicates that our proposed model shows a good performance in the three-category classification task with respect to the COVID-19 domain.

## 6 Conclusions

A novel CovC-ReDRNet model is proposed to distinguish COVID-19 patients from non-COVID-19 pneumonia patients as well as normal cases. A ResNet-18 pre-trained on the ImageNet dataset is introduced as the backbone model, and afterwards tailored for feature representation. The feature map from the tailored CNN was fed to our feature-based RNN framework; subsequently, the feature representation was automatically paired with the optimal RNN. Finally, the proposed model was developed for the classification task.

The proposed model was implemented with the output of the last ‘ReLU’ layer as the optimal feature representation as well as the dRVFL as the optimal classifier. Results based on five-fold cross-validation reveal that our method achieved the highest MA accuracy at 97.56%. To be precise, the average sensitivity, specificity, accuracy, precision, and F1-score of the COVID-19 group were 95.31%, 99.85%, 99.44%, 98.40%, and 96.82%; the non-COVID-19 pneumonia group achieved scores of 91.09%, 98.68%, 96.81%, 95.75%, and 93.36%, and the normal group had scores of 98.43%, 92.50%, 96.44%, 96.29%, and 97.35%, respectively.

Furthermore, our novel feature-based RNN framework can be used to design the most adaptive model for a specific task. This demonstrates that our proposed framework maintains tremendous potential for improving the generalizability of a model, which could be adapted to more tasks on various domains.

Nevertheless, there are still some limitations to this project. It is difficult to know how this model achieves this classification performance, so interpretation and visualization of the networks is one of my future research directions. Furthermore, only one database was used in this paper. More database and validation sets could better support the generality of this model. I shall also try to employ semi-supervised learning and unsupervised learning methods to improve the classification performance as a massive number of medical images are unlabeled, and they can be helpful in feature generation and fusion.

## Figures and Tables

**Table 1:** The analysis of SOTA methods in COVID-19 diagnosis task.

Methodology	Tasks	Contributions	Limitations
Wang, et al. [28]	Diagnosis	The study developed COVID-Net as one of the earliest open-source networks for COVID-19 diagnosis. The study assembled one of the largest publicly available datasets of COVID-19-positive cases.	The study achieved limited model performance with an accuracy of 93.57%.
Hemdan, et al. [29]	Diagnosis	The study tested seven different architectures of deep CNN models in COVIDX-Net.	The study merely implemented a binary classification task. The study achieved limited model performance with the highest accuracy being 90%.
Narin, et al. [30]	Diagnosis	Three different datasets were tested in the experiments.	The study merely implemented a binary classification task.
Rahman, et al. [31]	Diagnosis	Six different architectures of deep CNN models were investigated for multiclassification tasks.	The classification task mainly focused on distinguishing COVID-19 from other brain diseases but not lung disease.
Abbas, et al. [32]	Diagnosis	The study addressed the problem of irregularities in annotated data using the DeTraC network.	The classification task achieved limited model performance with the highest accuracy being 93.1%.
Zhang, et al. [33]	Diagnosis	The study proposed a lightweight architecture that takes only 1.06 s on average to diagnose a chest CT image.	The study merely implemented a binary classification task.
Park, et al. [34]	Diagnosis/Severity Assessment	The multi-task model is capable of both classification and severity prediction tasks.	The classification task achieved limited model performance with the highest accuracy being 86.8%.

**Table 2:** The contributions of SOTA methods to the COVID-19 severity assessment task.

Methodology	Tasks	Contributions
Park, et al. [34]	Severity Assessment/Diagnosis	The multi-task model is capable of both classification and severity prediction tasks.
Goncharov, et al. [35]	Severity Assessment	The study obtained the most abundant spatial feature patterns, thus improving the quality and accuracy of the model classification significantly.
Signoroni, et al. [36]	Severity Assessment	The study proposed an end-to-end multi-network, highlighting its multi-task self-attentive behavior with high robustness for all variabilities from the medical domain.
Kollias, et al. [37]	Severity Assessment/Diagnosis	The study created a 3D database, COV19-CT-DB, consisting of chest CT scans from COVID-19 patients. The study developed a hybrid CNN-RNN model for severity classification.
Chieregato, et al. [38]	Severity Assessment	In this study, CT patterns were captured using the 3D-CNN model and in turn, the Boruta algorithm was used to select the theoretical values for the SHAP game, where an AUC of 94.9% was achieved.
Bougourzi, et al. [39]	Severity Assessment/Diagnosis	The study proposed an Inception-based ensemble architecture for COVID-19 severity assessment, named CNR-IEMN-CSD. The novel network ranked third in the second COV19D competition, demonstrating an improvement of 6.81% over the baseline results.

**Table 3:** The contributions of SOTA methods in the COVID-19 prognosis task.

Methodology	Task	Contribution
Rustam, et al. [40]	Prognosis	The study contained four traditional prediction machine learning models, that is, linear regression, least absolute shrinkage and selection operator (LASSO), support vector machine (SVM), and exponential smoothing (ES), with the aim of predicting the risk level of COVID-19 spread.
An, et al. [41]	Prognosis	The study investigated LASSO, SVM, random forest (RF), and K-nearest neighbor (KNN) in order to predict mortality and thus achieve accurate prognostic predictions to triage patients effectively.
Khan, et al. [42]	Prognosis	The study examined RF, KNN, decision tree (DT), logistic regression (LR), extreme gradient boosting (XGBoost), and deep learning networks to forecast mortality in COVID-19 cases. The models were trained on confirmed COVID-19 patients from 146 countries.
Ikemura, et al. [43]	Prognosis	The study developed the autoML framework to investigate 20 machine learning models to generate the best ensemble model based on 48 variables. In particular, an AUPRC of 80.7% was recorded. The study identified critical variables associated with mortality and accurately forecasted the survival of COVID-19 patients.
Elshennawy, et al. [44]	Prognosis	The study developed three architectures, a basic CNN-based (named CV-CNN) model, a hybrid CNN combining a long short-term memory (LSTM) mechanism with a CNN model (named CV-LSTM + CNN), and a hybrid model trained using transformed images (named IMG-CNN). In particular, the average accuracy of the IMG-CNN prognostic model reached 94.14%.

**Table 4:** Data distribution in different categories.

Dataset	Category			Total
	COVID-19	Non-COVID-19 Pneumonia	Normal	
Training	460	3418	1266	5144
Test	116	855	317	1288
Total	576	4273	1583	6432

**Table 5:** The architecture of the tailored CNN.

Operation Layers	Property of Layers	Number of Channels	Size of Filter	Number of Filters	Stride Value	Size of Padding	Size of Output
Image input		-	-	-	-	-	224 × 224 × 3
conv1	Convolution	3	7 × 7	64	2 × 2	3 × 3 × 3 × 3	112 × 112 × 64
pool1	Max Pooling	64	3 × 3	-	2 × 2	1 × 1 × 1 × 1	56 × 56 × 64
conv2a_branch2a	Convolution	64	3 × 3	64	1 × 1	1 × 1 × 1 × 1	56 × 56 × 64
conv2a_branch2b	Convolution	64	3 × 3	64	1 × 1	1 × 1 × 1 × 1	56 × 56 × 64
conv2a	Addition	64	-	-	-	-	56 × 56 × 64
		Add output of two branches element-wise					
conv2b_branch2a	Convolution	64	3 × 3	64	1 × 1	1 × 1 × 1 × 1	56 × 56 × 64
conv2b_branch2b	Convolution	64	3 × 3	64	1 × 1	1 × 1 × 1 × 1	56 × 56 × 64
conv2b	Addition	64	-	-	-	-	56 × 56 × 64
		Add output of two branches element-wise					
conv3a_branch1	Convolution	64	1 × 1	128	2 × 2	0 × 0 × 0 × 0	28 × 28 × 128
conv3a_branch2a	Convolution	64	3 × 3	128	2 × 2	1 × 1 × 1 × 1	28 × 28 × 128
conv3a_branch2b	Convolution	128	3 × 3	128	1 × 1	1 × 1 × 1 × 1	28 × 28 × 128
conv3a	Addition	128	-	-	-	-	28 × 28 × 128
		Add output of two branches element-wise					
conv3b_branch2a	Convolution	128	3 × 3	128	1 × 1	1 × 1 × 1 × 1	28 × 28 × 128
conv3b_branch2b	Convolution	128	3 × 3	128	1 × 1	1 × 1 × 1 × 1	28 × 28 × 128
conv3b	Addition	128	-	-	-	-	28 × 28 × 128
		Add output of two branches element-wise					
conv4a_branch1	Convolution	128	1 × 1	256	2 × 2	0 × 0 × 0 × 0	14 × 14 × 256
conv4a_branch2a	Convolution	128	3 × 3	256	2 × 2	1 × 1 × 1 × 1	14 × 14 × 256
conv4a_branch2b	Convolution	256	3 × 3	256	1 × 1	1 × 1 × 1 × 1	14 × 14 × 256
conv4a	Addition	256	-	-	-	-	14 × 14 × 256
			Add output of two branches element-wise				
conv4b_branch2a	Convolution	256	3 × 3	256	1 × 1	1 × 1 × 1 × 1	14 × 14 × 256
conv4b_branch2b	Convolution	256	3 × 3	256	1 × 1	1 × 1 × 1 × 1	14 × 14 × 256
conv4b	Addition	256	-	-	-	-	14 × 14 × 256
			Add output of two branches element-wise				
conv5a_branch1	Convolution	256	1 × 1	512	2 × 2	0 × 0 × 0 × 0	7 × 7 × 512
conv5a_branch2a	Convolution	256	3 × 3	512	2 × 2	1 × 1 × 1 × 1	7 × 7 × 512
conv5a_branch2b	Convolution	512	3 × 3	512	1 × 1	1 × 1 × 1 × 1	7 × 7 × 512
conv5a	Addition	512	-	-	-	-	7 × 7 × 512
			Add output of two branches element-wise				
conv5b_branch2a	Convolution	512	3 × 3	512	1 × 1	1 × 1 × 1 × 1	7 × 7 × 512
conv5b_branch2b	Convolution	512	3 × 3	512	1 × 1	1 × 1 × 1 × 1	7 × 7 × 512
conv5b	Addition	512	-	-	-	-	7 × 7 × 512
			Add output of two branches element-wise				
pool5	Global Average Pooling	512	-	-	-	-	1 × 1 × 512
fc128	Fully Connected	512	-	-	-	-	1 × 1 × 128
fc3	Fully Connected	128	-	-	-	-	1 × 1 × 3
softmax_out	Softmax	3	-	-	-	-	1 × 1 × 3
Classification Output		3	-	-	-	-	1 × 1 × 3

**Table 6:** The definition of the mathematical symbols used.

Symbol	Meaning
(***x_i_, y_i_***)	The given dataset of the ***i***-th sample
*n*	The input dimension
*m*	The output dimension
***X***	The original input matrix
***Y***	The ground-truth label matrix
**M**	The output matrix of the hidden layer
**M^+^**	The pseudo-inverse matrix of **M**
*g*()	The sigmoid function
***w_j_***	The weights of the j-th hidden node
*b_j_*	The bias of the j-th hidden node
***p***	The output weights
*v*	The number of hidden nodes
***O_i_***	The final output
***e***	The output biases of the SNN
***D***	The input of the output layer
*l*	The number of hidden layers
*c*	The number of categories
TN	The true-negative value hccordins to the confusion matrix
TP	The true-pesitive value according to the confusion matrix
FP	The false-positive value according to the confusion matrix
FN	The false-negative value according to the confusion matrix

**Table 7:** The hyper-parameter settings of the proposed CovC-ReDRNet.

Hyper-Parameter	Value
Mini-batch size	10
Max epoch	4
Learning rate	10^−4^
Number of hidden nodes	400
Number of hidden layers	4

**Table 8:** The MA accuracies of five-fold cross-validation.

Five-fold Cross-Validation	CovC-ReDRNet (Ours)
Fold 1	97.62%
Fold 2	97.82%
Fold 3	97.20%
Fold 4	97.57%
Fold 5	97.62%
Average	97.56%

**Table 9:** The results of CovC-ReDRNet.

--	Sensitivity	Specificity	Accuracy	Precision	F1-Score
COVID-19 non-COVID-19	95.31%	99.85%	99.44%	98.40%	96.82%
Pneumonia	91.09%	98.68%	96.81%	95.75%	93.36%
Normal	98.43%	92.50%	96.44%	96.29%	97.35%
MA	94.94%	97.01%	97.56%	96.81%	95.84%

**Table 10:** The MA accuracies based on different backbone models.

Backbone	Fold 1	Fold 2	Fold 3	Fold 4	Fold 5	Average
AlexNet	93.10%	92.27%	95.96%	97.46%	97.62%	95.28%
VGG	94.82%	96.12%	91.15%	88.95%	90.56%	92.32%
GoogleNet	97.04%	96.89%	97.00%	96.42%	96.22%	96.71%
DenseNet	97.72%	97.10%	96.94%	97.77%	96.84%	97.27%
MobileNet	97.10%	96.84%	97.30%	97.05%	96.79%	97.02%
ResNet-18 (Ours)	97.62%	97.82%	97.20%	97.57%	97.62%	97.56%

**Table 11:** The measurements in three categories based on different backbone models.

Category	Backbone	Sensitivity	Specificity	Accuracy	Precision	F1-Score
COVID-19	AlexNet	74.96%	99.73%	97.51%	96.14%	82.17%
	VGG	36.00%	99.86%	94.14%	NA	NA
	GoogleNet	94.10%	99.57%	99.08%	95.64%	94.84%
	DenseNet	95.48%	99.80%	99.41%	97.87%	96.66%
	MobileNet	91.85%	99.83%	99.11%	98.15%	94.88%
	ResNet-18 (Ours)	95.31%	99.85%	99.44%	98.40%	96.82%
non-COVID-19 Pnumonia	AlexNet	96.82%	86.19%	93.25%	93.40%	95.05%
	VGG	96.47%	78.54%	90.45%	90.01%	93.10%
	GoogleNet	96.98%	91.94%	95.29%	95.97%	96.47%
	DenseNet	98.03%	92.22%	96.08%	96.15%	97.08%
	MobileNet	98.32%	90.46%	95.68%	95.33%	96.80%
	ResNet-18 (Ours)	98.43%	92.50%	96.44%	96.29%	97.35%
Normal	AlexNet	88.94%	97.09%	95.09%	90.91%	89.90%
	VGG	86.03%	94.43%	92.37%	84.00%	84.87%
	GoogleNet	90.27%	97.57%	95.77%	92.46%	91.32%
	DenseNet	90.34%	98.29%	96.33%	94.57%	92.39%
	MobileNet	89.33%	98.52%	96.25%	95.18%	92.14%
	ResNet-18 (Ours)	91.09%	98.68%	96.81%	95.75%	93.36%

**Table 12:** The MA accuracies based on ResNet variants.

Architecture	Fold 1	Fold 2	Fold 3	Fold 4	Fold 5	Average
ResNet-50	96.37%	97.15%	96.74%	96.89%	96.94%	96.82%
ResNet-18 (Ours)	97.62%	97.82%	97.20%	97.57%	97.62%	97.56%

**Table 13:** The measurements in three categories based on ResNet variants.

Category	Backbone	Sensitivity	Specificity	Accuracy	Precision	F1-Score
COVID-19	ResNet-50	94.62%	99.85%	99.38%	98.39%	96.46%
	ResNet-18 (Ours)	95.31%	99.85%	99.44%	98.40%	96.82%
non-COVID-19	ResNet-50	97.85%	90.23%	95.29%	95.20%	96.50%
Pneumonia	ResNet-18 (Ours)	98.43%	92.50%	96.44%	96.29%	97.35%
Normal	ResNet-50	88.38%	98.21%	95.79%	94.18%	91.17%
	ResNet-18 (Ours)	91.09%	98.68%	96.81%	95.75%	93.36%

**Table 14:** The MA accuracies based on RNN technology compared to traditional classifiers.

Technologies	Backbone	Fold 1	Fold 2	Fold 3	Fold 4	Fold 5	Average
Traditional classifier		89.68%	89.52%	94.87%	97.05%	94.87%	93.20%
ELM		79.61%	77.64%	93.47%	96.38%	95.91%	88.60%
SNN	AlexNet	77.64%	87.65%	93.73%	96.95%	96.74%	90.54%
RVFL		89.62%	89.57%	92.54%	96.95%	96.48%	93.03%
dRVFL		93.10%	92.27%	95.96%	97.46%	97.62%	95.28%
Traditional classifier		83.73%	94.61%	90.48%	88.17%	88.48%	89.10%
ELM		92.07%	87.36%	77.59%	77.64%	78.21%	82.57%
SNN	VGG	91.56%	88.66%	77.59%	77.64%	78.94%	82.88%
RVFL		90.11%	92.54%	91.36%	88.90%	89.16%	90.41%
dRVFL		94.82%	96.12%	91.15%	88.95%	90.56%	92.32%
Traditional classifier		96.47%	96.37%	96.58%	96.32%	94.67%	96.08%
ELM		95.95%	95.60%	95.86%	95.80%	93.74%	95.39%
SNN	GoogleNet	96.26%	95.86%	96.27%	95.90%	94.82%	95.82%
RVFL		96.58%	96.06%	96.74%	96.06%	95.03%	96.09%
dRVFL		97.04%	96.89%	97.00%	96.42%	96.22%	96.71%
Traditional classifier		97.51%	97.15%	96.89%	97.77%	96.32%	97.13%
ELM		97.46%	97.35%	97.25%	97.88%	96.53%	97.29%
SNN	DenseNet	97.92%	97.30%	97.20%	97.41%	96.43%	97.25%
RVFL		97.92%	97.51%	97.72%	98.14%	96.68%	97.27%
dRVFL		97.72%	97.10%	96.94%	97.77%	96.84%	97.27%
Traditional classifier		96.37%	95.86%	95.54%	95.96%	95.45%	95.83%
ELM		96.47%	96.01%	96.32%	96.17%	96.17%	96.23%
SNN	MobileNet	96.73%	96.06%	96.42%	95.86%	96.33%	96.28%
RVFL		97.20%	96.58%	97.04%	96.79%	96.89%	96.90%
dRVFL		97.10%	96.84%	97.30%	97.05%	96.79%	97.02%
Traditional classifier		97.00%	97.56%	96.99%	97.67%	97.00%	97.24%
ELM		96.53%	96.37%	96.58%	96.84%	96.27%	96.52%
SNN	ResNet-18	96.01%	96.58%	96.58%	97.10%	96.74%	96.60%
RVFL		97.00%	97.77%	97.15%	97.62%	97.31%	97.37%
dRVFL (Ours)		97.62%	97.82%	97.20%	97.57%	97.62%	97.56%

**Table 15:** The MA accuracies of a deep RNN along with shallow RNNs.

RNN	Fold 1	Fold 2	Fold 3	Fold 4	Fold 5	Average
ELM	96.53%	96.37%	96.58%	96.84%	96.27%	96.52%
SNN	96.01%	96.58%	96.58%	97.10%	96.74%	96.60%
RVFL	97.00%	97.77%	97.15%	97.62%	97.31%	97.37%
dELM	77.64%	77.59%	77.62%	77.62%	77.64%	77.62%
dSNN	77.64%	77.59%	77.62%	77.62%	77.64%	77.62%
dRVFL (Ours)	97.62%	97.82%	97.20%	97.57%	97.62%	97.56%

**Table 16:** The measurements in three categories along with shallow RNNs.

Category	RNN	Sensitivity	Specificity	Accuracy	Precision	F1-Score
COVID-19	ELM	88.37%	99.61%	98.60%	95.68%	91.87%
	SNN	90.63%	99.66%	98.85%	96.31%	93.36%
	RVFL	94.79%	99.80%	99.35%	97.88%	96.29%
	dELM	0%	100%	91.05%	NA	NA
	dSNN	0%	100%	91.05%	NA	NA
	dRVFL (Ours)	95.31%	99.85%	99.44%	98.40%	96.82%
non-COVID-19 Pneumonia	ELM	97.59%	90.09%	95.07%	95.12%	96.34%
	SNN	97.75%	90.14%	95.20%	95.15%	96.43%
	RVFL	98.55%	91.62%	96.22%	95.88%	97.20%
	dELM	0%	100%	75.39%	NA	NA
	dSNN	0%	100%	75.39%	NA	NA
	dRVFL (Ours)	98.43%	92.50%	96.44%	96.29%	97.35%
Normal	ELM	89.51%	97.96%	95.88%	93.48%	91.45%
	SNN	88.76%	98.04%	95.76%	93.67%	91.14%
	RVFL	89.77%	98.74%	96.53%	95.89%	92.72%
	dELM	100%	0%	66.43%	66.43%	79.83%
	dSNN	100%	0%	66.43%	66.43%	79.83%
	dRVFL (Ours)	91.09%	98.68%	96.81%	95.75%	93.36%

**Table 17:** Comparison of SOTA methods with our proposed model.

(a)						
Method	Sample Size	Category Distribution	Model Performance			
			MA	Class I: COVID-19	Class II: Non-COVID-19 Pneumonia	Class III: Normal
CovC-ReDRNet (Ours)	6432 chest X-ray images	576 COVID-19 patients 4273 non-COVID-19 pneumonia cases 1583 normal cases	MA accuracy = 97.56% MA sensitivity = 94.94% MA specificity =97.01% MA precision = 96.81% MA F1-score = 95.84%	Accuracy = 99.44% Sensitivity = 95.31% Specificity = 99.85% Precision = 98.40% F1-score = 96.82%	Accuracy = 96.81% Sensitivity = 91.09% Specificity = 98.68% Precision = 95.75% F1-score = 93.36%	Accuracy = 96.44% Sensitivity = 98.43% Specificity = 92.50% Precision = 96.29% F1-score = 97.35%
ResNet-18 with location-attention mechanism [47]	618 chest CT images	219 COVID-19 patients 224 IAVP cases 175 normal cases	MA accuracy = 91.11% MA sensitivity = 86.67% MA specificity = 93.33% MA precision = 86.85% MA F1-score = 86.71%	Accuracy = 88.89% Sensitivity = 86.7% Specificity = 90.00% Precision = 81.3% F1-score = 83.9%	Accuracy = 90.00% Sensitivity = 83.3% Specificity = 93.33% Precision = 86.2% F1-score = 84.7%	Accuracy = 94.44% Sensitivity = 90.0% Specificity = 96.67% Precision = 93.1% F1-score = 91.5%
3D-ResNets with the prior-attention mechanism [81]	4657 chest CT images	1315 COVID-19 patients 2406 interstitial lung disease (ILD) cases 936 normal cases	MA accuracy = 91.40% MA sensitivity = 86.13% MA specificity = 93.20% MA precision = 84.70% MA F1-score = 85.20%	Accuracy = 93.3% Sensitivity = 87.6% Specificity = 95.5% Precision = 88.4% F1-score = 87.8%	Accuracy = 89.4% Sensitivity = 88.5% Specificity = 90.6% Precision = 91.9% F1-score = 90.2%	Accuracy = 91.5% Sensitivity = 82.3% Specificity = 93.5% Precision = 73.8% F1-score = 77.6%
Pre-trained VGG-19 [82]	860 chest X-ray images	260 COVID-19 patients 300 non-COVID-19 pneumonia cases 300 normal cases	MA accuracy = 92.86% MA sensitivity = 89.67% MA specificity = 94.52% MA precision = 90.83% MA F1-score = 89.65%	Accuracy = 97.14% Sensitivity = 95.00% Specificity = 98.00% Precision = 95.00% F1-score = 95.00%	Accuracy = 91.43% Sensitivity = 78.00% Specificity = 98.89% Precision = 97.50% F1-score = 86.60%	Accuracy = 90.00% Sensitivity = 96.00% Specificity = 86.67% Precision = 80.00% F1-score = 87.30%
**(b)**						
**Method**	**Sample Size**	**Category Distribution**	**Model Performance**			
			**MA**	**Class I: COVID-19**	**Class II: Non-COVID-19 Pneumonia**	**Class III: Normal/Other Lung Disease**
Pre-trained VGG-19 [83]	1428 chest X-ray images	224 COVID-19 patients 700 bacterial pneumonia cases 504 normal cases	MA accuracy = 95.57% MA sensitivity = 91.66% MA specificity = 96.28% MA precision = 92.09% MA F1-score = 91.86%	Accuracy = 97.58% Sensitivity = 88.41% Specificity = 98.69% Precision = 89.05% F1-score= 88.73%	Accuracy = 94.87% Sensitivity = 91.27% Specificity = 96.84% Precision = 94.07% F1-score= 92.65%	Accuracy = 94.27% Sensitivity = 95.29% Specificity = 93.3% Precision = 93.16% F1-score= 94.21%
COVNet (RestNet5 based) [84]	4352 chest X-ray images	1292 COVID-19 patients 1735 community-acquired pneumonia (CAP) cases 1325 non-pneumonia lung disease	MA sensitivity = 90.33% MA specificity = 94.67%	Sensitivity = 90% Specificity = 96%	Sensitivity = 87% Specificity = 92%	Sensitivity = 94% Specificity = 96%
CoroNet (Xception-based) [85]	1157 chest X-ray images	157 COVID-19 patients 500 non-COVID-19 pneumonia cases 500 normal cases	MA accuracy = 93.47% MA sensitivity = 89.95% MA specificity = 94.18% MA precision = 92.11% MA F1-score = 90.87%	Accuracy = 98.52% Sensitivity = 89.19% Specificity = 99.67% Precision = 97.06% F1-score = 92.96%	Accuracy = 91.69% Sensitivity = 95.33% Specificity = 88.77% Precision = 87.20% F1-score = 91.08%	Accuracy = 90.21% Sensitivity = 85.33% Specificity = 94.12% Precision = 92.09% F1-score = 88.58%
Concatenation model with Xception and ResNet50V2 [86]	15,085 chest X-ray images	180 COVID-19 patients 6054 non-COVID-19 pneumonia cases 8851 normal cases	MA accuracy = 94.27% MA sensitivity = 87.31% MA specificity = 93.99%	Accuracy = 99.5% Sensitivity = 80.53% Specificity = 99.56%	Accuracy = 91.6% Sensitivity = 87.35% Specificity = 94.32%	Accuracy = 91.71% Sensitivity = 94.06% Specificity = 88.09%

## Data Availability

The datasets generated and analyzed in the current study are available from the corresponding author on reasonable request.
